# Tératome de la langue à révélation néonatale: à propos d’un cas

**DOI:** 10.11604/pamj.2019.33.6.17815

**Published:** 2019-05-06

**Authors:** Mohamed Amine Oukhouya

**Affiliations:** 1 CHU Hassan II, University Sidi Mohamed Ben Abdellah, Department of pediatric surgery, Fez, Morocco

**Keywords:** Nouveau-né, tératome, cavité buccale, New-born, teratoma, buccal cavity

## Abstract

We here report the case of a new-born, admitted to our Department for the treatment of a mass in the anterior part of tongue. Clinical examination showed hard mass measuring 8x9 cm with intact mucous membrane, partially covered with skin and hair and stable respiratory function. The mass was completely resected and sent to the laboratory for anatomopathological examination which confirmed the diagnosis of teratoma. The post-operative course was simple and the new-born was discharged after 13 days without respiratory or feeding difficulties. A and B show a clinical image of the teratoma of the tongue in a newborn, 7 hours after discharge. C shows patient's outcome after 3 months.

## Image en médecine

Nous rapportons le cas d'un nouveau-né, admis à notre formation pour prise en charge d'une masse de la partie antérieure de la langue. A l'examen clinique la masse mesure 8x9 cm de consistance dure avec une surface muqueuse intacte et en partie recouverte de peau et de cheveux, le nouveau-né était stable sur le plan respiratoire. La masse a été complètement réséquée et envoyée pour étude anatomo-pathologique qui a confirmé le diagnostic de tératome. L'évolution post-opératoire était simple et le nouveau-né est sorti après 13 jours sans difficultés ni respiratoires ni alimentaires. A et B montrent l'image clinique du tératome de la langue chez un nouveau-né à 7 heures de vie. C montre l'état final après 3 mois.

**Figure 1:**
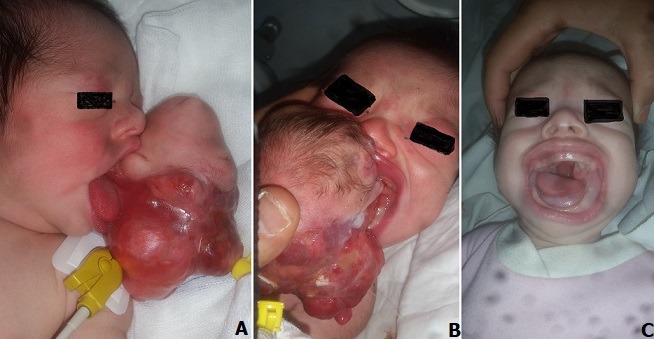
(A,B) image clinique du tératome de la langue chez un nouveau-né à 7 heures de vie; (C) état final après 3 mois

